# Assessing the health of the general population in England: how do the three- and five-level versions of EQ-5D compare?

**DOI:** 10.1186/s12955-015-0356-8

**Published:** 2015-10-21

**Authors:** Yan Feng, Nancy Devlin, Mike Herdman

**Affiliations:** Office of Health Economics, 7th Floor, Southside, 105 Victoria Street, SW1E 6QT, London, UK

**Keywords:** EQ-5D-5L, EQ-5D-3L, Population Survey, England

## Abstract

**Background:**

The EQ-5D is a brief, generic measure of health status that can be easily incorporated into population health surveys. There are two versions of the EQ-5D for use in adult populations, one with 3 response levels in each of the instrument’s 5 dimensions (EQ-5D-3L) and one with 5 levels in each dimension (EQ-5D-5L). We compared the two versions as measures of self-reported health status in representative samples of the English general population.

**Methods:**

EQ-5D-5L data were available from 996 respondents selected at random from residential postcodes who took part in the EQ-5D-5L value set for England study. EQ-5D-3L data were available from 7294 participants included in the 2012 Health Survey for England. Responses on the 3L and 5L versions of EQ-5D were compared by examining score distributions on the two versions, both in terms of the profile (dimensions) and the EQ-VAS. To determine the extent of variations in score according to respondent characteristics, we analysed health status reporting on the descriptive profile, EQ-5D Index, and EQ-VAS of both versions of EQ-5D by age, sex, and educational background. We used *X*^2^ to test for differences between respondent categories when analyzing EQ-5D profile data and the *t* test when analyzing EQ-5D Index and VAS scores.

**Results:**

The 5L version of EQ-5D led to a considerably reduced ceiling effect and a larger proportion of respondents reporting severe health problems compared to the 3L. The 5L version also led to the use of a wider spread of health states; just 3 health states on the 3L covered 75 % of the sample, compared to 12 states on the 5L. Both versions showed poorer health status in older respondents, females, and those in a lower educational category and the EQ-5D-5L descriptive system, though not the Index or VAS, discriminated better between age groups than the 3L. There were no appreciable differences between the two versions in their ability to discriminate between groups defined by gender or educational level.

**Conclusions:**

The new, expanded 5L version of EQ-5D may be a more useful instrument for the measurement of health status in population health surveys than the original 3L version.

## Background

Measuring self-reported health status is an important part of many population health surveys. As noted on the NHS Health Scotland website “describing and understanding the health of [a] population and the factors that shape it is essential to improving health and reducing inequalities. It enables good design of actions, targeting of resources and assessment of the impact of programmes and policies” [1]. Assessing health status in large-scale population surveys can help identify groups within the population which require particular attention and, if performed on a regular basis, can show how population health evolves over time. Comparing health status across countries can also be of interest [2].

The EQ-5D is a widely used measure of health status which has been included in several population health surveys [3, 4]. It comprises a descriptive system which assesses health in 5 dimensions (mobility, self-care, usual activities, pain/discomfort, anxiety/depression) and a Visual Analog Scale (VAS) on which the respondent rates their overall health on the day of completion. In the original version of the instrument, each dimension in the descriptive system is assessed using 3 levels of severity [5]. In order to reduce high ceiling effects (i.e. the proportion of respondents reporting the best possible health on EQ-5D who are therefore unable to record any improvement in health status) reported in some populations [6–12], and to increase the instrument’s sensitivity to changes in health, a new version of the instrument was developed using the same 5 dimensions, but with 5 levels of severity in each [13]. Studies in Germany [14] and South Korea [15] indicated that the ceiling effect was reduced in the 5L version but, as far as we are aware, only Craig et al. (2014) directly compared the performance of the 3L and 5L versions of EQ-5D in a general population sample [16]. They found fewer ceiling effects with the 5L and therefore a greater frequency of health problems. On the other hand, they suggested that the health problems were less severe with the 5L compared to the 3L, particularly in the pain/discomfort and anxiety/depression dimensions [16].

The aim of the present study was to compare the performance of the 3L and 5L versions of EQ-5D in representative samples of the English general public.

## Methods

### Data and sampling approach

We used two datasets in this study. Self-reported health data on the EQ-5D-5L was obtained from participants in the value set study for England [17] while general population data for the 3L was obtained from the 2012 Health Survey for England (HSE) [18]. In both cases, EQ-5D data were collected in face-to-face, computer assisted interviews.

As well as respondents’ self-reported health status on the EQ-5D dimensions (EQ profiles), both datasets also included respondents’ self-reported EQ-VAS scores, demographic characteristics (sex, age and health limits), and socio-economic characteristics (employment status, retirement status, education background, and religion).

EQ-5D-5L data were available from 996 participants selected at random from residential postcodes. The sample was intended to be representative of adults aged 18 years and over living in private residential accommodation in England. Individuals living in communal establishments were excluded. Respondents were interviewed between November 2012 and March 2013. A sample of 2020 addresses from 66 primary sampling units (based on postcode sectors) across England was randomly selected, using the Post Office small user Postcode Address File (PAF) as the sampling frame. A total of 1004 individuals were interviewed. Their self-reported EQ-5D-5L data were collected prior to the valuation task in which they were asked to value EQ-5D health states, and were recorded using an electronic data capture system (EQ-VT). The household response rate was approximately 50 % [17].

EQ-5D-3L data were available from the 2012 HSE. The survey covered the adult population aged 16 years and over living in private households in England and provided a representative sample of the population at both national and regional level. 9024 addresses were randomly selected in 564 postcode sectors. Respondents were interviewed between January 2012 and December 2012. Where an address was found to have multiple dwelling units, a random selection was made and a single dwelling unit was included. Where there were multiple households at a dwelling unit, one was again selected at random. A total of 8291 adults and 2043 children were interviewed. A household response rate of 64 % was achieved [19] and the final dataset included self-reported health on EQ-5D-3L from 7294 respondents.

### EQ-5D-5L and EQ-5D-3L

The EQ-5D is a generic preference-based instrument that is widely used to measure and value changes in health-related quality of life [20]. For example, the 3L version of the instrument is being used throughout the English NHS as part of the Patients Reported Outcome Measures (PROMs) programme [21].

The EQ-5D instrument compromises two parts. In the first part, respondents describe their health status on the day of administration by checking one level of severity on each of the instrument’s five dimensions. The 3L version has three response levels in each dimension (none, some and extreme/unable to) while the 5L version has five levels (none, slight, moderate, severe, extreme/unable to). When developing the 5L version, some generally minor changes were made to wording. A more important change was the move from ‘I am confined to bed’ as the extreme level of mobility in the 3L to ‘I am unable to walk about’ in the 5L. This was done to make the mobility dimension more consistent with wording in other dimensions. The descriptive systems of the EQ-5D-5L and EQ-5D-3L are shown in Appendix 1.

In the second part of the instrument, respondents indicate how good or bad their health is on the day of administration on a health thermometer (EQ-VAS) which is presented as a 0–100, hash-marked, numbered vertical line with anchors of best and worst imaginable health state (100 and 0, respectively). The EQ-VAS is used to assess the overall health of respondents rather than selected dimensions of individuals’ health and there are slight differences in format and instructions between the 5L and 3L versions (see Appendix 2). One of the most important differences between the EQ-VAS in the two versions is that in the 3L respondents draw a line from a box labelled ‘Your own health today’ to a point on the scale which reflects their health on the day of the interview. In the 5L version, they are asked to mark an ‘x’ on the scale to indicate that point and then record their answer in the box provided.

One of the uses of the EQ-5D is to provide societal values (utilities) for health states generated by the instrument which can then be used in economic evaluations of health care interventions. These values are known as the EQ Index [22]. The 3L version of the instrument generates 243 possible health states (3^5^) compared to 3125 possible health states (5^5^) generated by the 5L. In order to calculate the EQ Index for the 3L version, we used the algorithm provided by [23], though it should be noted that the values used to construct that algorithm were for the UK, and not for England alone. Currently, no values are available for the UK for the 5L version of EQ-5D, so the Index score for that version was calculated using a crosswalk system from 3L values, as described in [24].

### Statistical analysis

The two datasets were compared to determine whether the two samples had similar demographic and socio-economic characteristics (Table 1).

**Table 1:** Demographic and socio-economic characteristics of the two study samples

Respondent characteristics	EQ-5D-5L valuation study (*n* = 996)	HSE 2012 (*n* = 7294)	*X* ^2^ (DF)	*P* value
Sex			*X* ^2^ (1) = 4.9	0.03
Female	591 (59.3 %)	4058 (55.6 %)		
Male	405 (40.7 %)	3236 (44.4 %)		
Age			*X* ^2^ (3) = 9.6	0.02
Below 35	202 (20.3 %)	1769 (24.3 %)		
35–54	381 (38.3 %)	2522 (34.6 %)		
55–64	155 (15.6 %)	1173 (16.1 %)		
65 and above	258 (25.9 %)	1830 (25.1 %)		
Employment status			*X* ^2^ (1) = 2.7	0.10
Yes	504 (50.6 %)	3893 (53.4 %)		
No/retired/economically inactive/no answer	492 (49.4 %)	3401 (46.6 %)		
Retirement status			*X* ^2^ (1) = 1.1	0.30
Yes	277 (27.8 %)	1915 (26.3 %)		
No or no answer	719 (72.2 %)	5379 (73.8 %)		
Health Limit ^a^			*X* ^2^ (1) = 7.0	0.00
Yes	268 (26.9 %)	1675 (23.0 %)		
No or no answer	728 (73.1 %)	5619 (77.0 %)		
Ethnic group			*X* ^2^ (1) = 0.1	0.80
White	899 (90.3 %)	6565 (90.0 %)		
Other or no answer	97 (9.7 %)	729 (10.0 %)		
Education background			*X* ^2^ (1) = 9.6	0.00
Degree or above	211 (21.2 %)	1877 (25.7 %)		
Other	785 (78.8 %)	5417 (74.3 %)		
Religion			*X* ^2^ (1) = 2.3	0.13
Christian	636 (63.9 %)	4477 (61.4 %)		
Other or no answer	360 (36.1 %)	2817 (38.6 %)		

^a^Defined as long lasting illness in HSE 2012

*X*^2^ tests showed that there were statistically significant differences between the two samples (*P* < 0.05) with the 5L sample having a slightly higher proportion of females, more respondents in the 35–54 age group and fewer in the youngest age group, and a slightly lower level of education.

Responses on the 3L and 5L versions of EQ-5D were compared by examining score distributions on the two versions, both in terms of the profile (dimensions) and the EQ-VAS. This analysis included examination of ceiling and floor effects, i.e., respondents reporting the best and worst health states on the two versions, i.e. state 11111 (best) and state 33333 (worst 3L state) or 55555 (worst 5L state). For the EQ profile, we estimated the proportion of patients reporting problems on each level in each dimension and listed all health states reported in order of frequency. We estimated the top 10 most frequently self-reported health states on both versions. EQ-VAS data was analysed using a similar approach, by calculating the 10 most frequently self-reported scores on the 3L and 5L versions of EQ-VAS, the frequency of those scores, and the proportion of total sample size they represented.

To determine the extent of variations in score according to respondent characteristics, we analysed health status reporting on the descriptive profile, EQ-5D Index, and EQ-VAS of both versions of EQ-5D by age (i.e. under 35, 35–54, 55–64, ≥65 years), sex, and educational background (i.e. respondents with a degree vs those without). We expected that older respondents, females, and those in lower educational categories would report poorer health status [2, 9, 12], but also aimed to determine whether there were any differences between the 3L and 5L in terms of their ability to discriminate between socio-demographic groups known to differ in health status, i.e. between younger and older respondents, between men and women, and between those with higher and lower levels of education. We used the *X*^2^ test to check for differences between respondent categories when analyzing EQ-5D profile data. Specifically, we tested for differences between the two versions of the EQ-5D in the proportion of respondents self-reporting health state 11111, the proportion of respondents self-reporting level 1 in each dimension, and the proportions of respondents reporting poor health (level 3 for the 3L instrument and levels 4 and 5 for the 5L instrument), by age, sex and educational background. We used the *t* test in a similar analysis of EQ-5D Index and VAS scores. Although neither the Index nor the VAS showed a normal distribution, we decided to use a parametric test of differences because of the large sample size, the fact that non-parametric tests require similar variance in all samples (which was not the case here) and because parametric tests have more statistical power than non-parametric tests, and are therefore more likely to detect significant differences between samples. Statistical significance was set at *P* < 0.05 for all tests. All analyses were performed in STATA/MP 12.1.

## Results

Score distributions on the descriptive systems of the two versions are shown in Table 2 for the overall samples.

**Table 2:** Distribution of responses on the 5L and 3L versions of EQ-5D; n (%)

EQ-5D-5L (*n* = 996)		EQ-5D-3L (*n* = 7294)	
Mobility			
Level 1	737 (74.0 %)	Level 1	6021 (82.6 %)
Level 2	113 (11.4 %)		
Level 3	80 (8.0 %)	Level 2	1260 (17.3 %)
Level 4	58 (5.8 %)		
Level 5	8 (0.8 %)	Level 3	13 (0.2 %)
Any problem in mobility	26.0 %	Any problem in mobility	17.5 %
Self care			
Level 1	904 (90.8 %)	Level 1	6894 (94.5 %)
Level 2	35 (3.5 %)		
Level 3	36 (3.6 %)	Level 2	377 (5.2 %)
Level 4	15 (1.5 %)		
Level 5	6 (0.6 %)	Level 3	23 (0.3 %)
Any problem in self care	9.2 %	Any problem in self care	5.5 %
Usual activities			
Level 1	760 (76.3 %)	Level 1	6130 (84.0 %)
Level 2	107 (10.7 %)		
Level 3	68 (6.8 %)	Level 2	1056 (14.5 %)
Level 4	49 (4.9 %)		
Level 5	12 (1.2 %)	Level 3	108 (1.5 %)
Any problem in usual activities	23.7 %	Any problem in usual activities	16.0 %
Pain/discomfort			
Level 1	582 (58.4 %)	Level 1	4848 (66.5 %)
Level 2	226 (22.7 %)		
Level 3	104 (10.4 %)	Level 2	2161 (29.6 %)
Level 4	71 (7.1 %)		
Level 5	13 (1.3 %)	Level 3	285 (3.9 %)
Any problem in pain/discomfort	41.6 %	Any problem in pain/discomfort	33.5 %
Anxiety/depression			
Level 1	757 (76.0 %)	Level 1	5831 (79.9 %)
Level 2	137 (13.8 %)		
Level 3	73 (7.3 %)	Level 2	1301 (17.8 %)
Level 4	20 (2.0 %)		
Level 5	9 (0.9 %)	Level 3	162 (2.2 %)
Any problem in anxiety/depression	24.0 %	Any problem in anxiety/depression	20.1 %

The 3L version of EQ-5D showed higher ceiling effects in all dimensions with, for example, 84 % of respondents reporting no problems with Usual Activities on the 3L compared to 76.3 % on the 5L. At the other end of the scale, we found that the 5L identified more respondents with serious health problems than the 3L (if we assume levels 4 and 5 on the 5L represent serious health problems). For example, 6.1 % of respondents reported serious problems with Usual Activities on the 5L compared to only 1.5 % on the 3L. A similar pattern was seen across the other dimensions. On both versions of EQ-5D, the proportion of respondents reporting problems decreased almost monotonically with increasing severity of the response options.

The ten most frequently observed self-reported health states on the 5L and 3L descriptive systems are shown in Table 3, together with the prevalence of the worst health state for each version. Respondents used a larger number of health states in the 5L than the 3L. In the 3L version, the cumulative frequency of the top 10 most frequently observed health states was just under 90 %. The remaining 10 % of observations were distributed over 88 health states. The most frequently observed self-reported health states showed a similar pattern across the two versions of the instrument with the best possible health state, 11111, accounting for 47.6 % of observations on the 5L and 56.2 % on the 3L, followed by health states representing mild/moderate levels of pain/discomfort and anxiety/depression, i.e. health states 11121 and 11112 (it should be noted that, apart from full health 11111, the same health state descriptors, e.g. 11112, do not represent the same level of problems on the 3L as on the 5L, as a 2, for example, represents ‘slight’ problems on the 5L but ‘some’ problems on the 3L). The prevalence of the worst possible health state is the lowest (0.05 %) among all checked health states (98 out of 243) in the 3L data, while none of the respondents reported the worst possible health state (55555) on the 5L. Respondents reported a greater range of health states on the 5L than on the 3L; the three most frequently observed health states accounted for almost 75 % of respondents on the 3L whilst a similar proportion of respondents on the 5L were accounted for by 12 health states. Of course, the number of available health states is much larger on the 5L than on the 3L (3125 vs 243).

**Table 3:** Prevalence of the 10 most frequently observed self-reported health states and frequency of reporting of the worst possible health states in EQ-5D-5L and EQ-5D-3L

EQ-5D-5L			EQ-5D-3L		
Health states	Frequency (%)	Cumulative frequency in %	Health states	Frequency (%)	Cumulative frequency (%)
11111	474 (47.6)	47.6	11111	4096 (56.2)	56.2
11121	93 (9.3)	56.9	11121	855 (11.7)	67.9
11112	46 (4.6)	61.6	11112	496 (6.8)	74.7
11131	22 (2.2)	63.8	11122	241 (3.3)	78.0
21121	21 (2.1)	65.9	21221	224 (3.1)	81.0
11122	21 (2.1)	68.0	21121	222 (3.0)	84.1
21221	19 (1.9)	69.9	21222	138 (1.9)	86.0
11123	13 (1.3)	71.2	11221	103 (1.4)	87.4
21111	11 (1.1)	72.3	11222	67 (0.9)	88.3
11221	11 (1.1)	73.4	22221	64 (0.9)	89.2
…			…		
55555	0 (0.0)	100	33333	4 (0.1)	100

Table 4 shows the prevalence of problems reported on the EQ-5D profile by age group. On both versions of EQ-5D, older respondents reported poorer health status. The proportion of respondents self-reporting their EQ-5D profile as 11111 was lower in all age groups using the 5L (*P* < 0.05) as was the proportion of respondents reporting no problems in each individual dimension (*P* < 0.05). This reduction in the ceiling effect with the 5L was particularly noticeable in the older age groups.

**Table 4:** Number of respondents (percentages) at different levels in the EQ-5D-5L profile and EQ-5D-3L profile by age groups

	Below 35 years old				35–54 years old				55–64 years old				65 years old and above			
Dimensions	5L		3L		5L		3L		5L		3L		5L		3L	
Mobility	1	185 (91.6 %)	1	1689 (95.5 %)	1	322 (84.5 %)	1	2235 (88.6 %)	1	102 (65.8 %)	1	908 (77.4 %)	1	128 (49.6 %)	1	1189 (65.0 %)
	2	8 (4.0 %)			2	31 (8.1 %)			2	25 (16.1 %)			2	49 (19.0 %)		
	3	6 (3.0 %)	2	76 (4.3 %)	3	17 (4.5 %)	2	283 (11.2 %)	3	15 (9.7 %)	2	261 (22.3 %)	3	42 (16.3 %)	2	640 (35.0 %)
	4	3 (1.5 %)			4	10 (2.6 %)			4	12 (7.7 %)			4	33 (12.8 %)		
	5	0 (0.0 %)	3	4 (0.2 %)	5	1 (0.3 %)	3	4 (0.2 %)	5	1 (0.7 %)	3	4 (0.3 %)	5	6 (2.3 %)	3	1 (0.1 %)
Self-care	1	197 (97.5 %)	1	1751 (99.0 %)	1	362 (95.0 %)	1	2426 (96.2 %)	1	136 (87.7 %)	1	1084 (92.4 %)	1	209 (81.0 %)	1	1633 (89.2 %)
	2	4 (2.0 %)			2	7 (1.8 %)			2	6 (3.9 %)			2	18 (7.0 %)		
	3	0 (0.0 %)	2	17 (1.0 %)	3	7 (1.8 %)	2	88 (3.5 %)	3	9 (5.8 %)	2	83 (7.1 %)	3	20 (7.8 %)	2	189 (10.3 %)
	4	1 (0.5 %)			4	5 (1.3 %)			4	3 (1.9 %)			4	6 (2.3 %)		
	5	0 (0.0 %)	3	1 (0.1 %)	5	0 (0.0 %)	3	8 (0.3 %)	5	1 (0.7 %)	3	6 (0.5 %)	5	5 (1.9 %)	3	8 (0.4 %)
Usual Activities	1	183 (90.6 %)	1	1653 (93.4 %)	1	326 (85.6 %)	1	2204 (87.4 %)	1	109 (70.3 %)	1	941 (80.2 %)	1	142 (55.0 %)	1	1332 (72.8 %)
	2	9 (4.5 %)			2	26 (6.8 %)			2	19 (12.3 %)			2	53 (20.5 %)		
	3	6 (3.0 %)	2	110 (6.2 %)	3	16 (4.2 %)	2	290 (11.5 %)	3	15 (9.7 %)	2	215 (18.3 %)	3	31 (12.0 %)	2	441 (24.1 %)
	4	3 (1.5 %)			4	11 (2.9 %)			4	9 (5.8 %)			4	26 (10.1 %)		
	5	1 (0.5 %)	3	6 (0.3 %)	5	2 (0.5 %)	3	28 (1.1 %)	5	3 (1.9 %)	3	17 (1.5 %)	5	6 (2.3 %)	3	57 (3.1 %)
Pain/Discomfort	1	153 (75.7 %)	1	1502 (84.9 %)	1	250 (65.6 %)	1	1810 (71.8 %)	1	76 (49.0 %)	1	651 (55.5 %)	1	103 (39.9 %)	1	885 (48.4 %)
	2	30 (14.9 %)			2	79 (20.7 %)			2	44 (28.4 %)			2	73 (28.3 %)		
	3	13 (6.4 %)	2	254 (14.4 %)	3	24 (6.3 %)	2	638 (25.3 %)	3	20 (12.9 %)	2	446 (38.0 %)	3	47 (18.2 %)	2	823 (45.0 %)
	4	5 (2.5 %)			4	22 (5.8 %)			4	12 (7.7 %)			4	32 (12.4 %)		
	5	1 (0.5 %)	3	13 (0.7 %)	5	6 (1.6 %)	3	74 (2.9 %)	5	3 (1.9 %)	3	76 (6.5 %)	5	3 (1.2 %)	3	122 (6.7 %)
Anxiety/Depression	1	167 (82.7 %)	1	1486 (84.0 %)	1	291 (76.4 %)	1	1970 (78.1 %)	1	111 (71.6 %)	1	913 (77.8 %)	1	188 (72.9 %)	1	1462 (79.9 %)
	2	21 (10.4 %)			2	57 (15.0 %)			2	25 (16.1 %)			2	34 (13.2 %)		
	3	8 (4.0 %)	2	256 (14.5 %)	3	22 (5.8 %)	2	476 (18.9 %)	3	11 (7.1 %)	2	226 (19.3 %)	3	32 (12.4 %)	2	343 (18.7 %)
	4	4 (2.0 %)			4	9 (2.4 %)			4	5 (3.2 %)			4	2 (0.8 %)		
	5	2 (1.0 %)	3	27 (1.5 %)	5	2 (0.5 %)	3	76 (3.0 %)	5	3 (1.9 %)	3	34 (2.9 %)	5	2 (0.8 %)	3	25 (1.4 %)
Sub-total	202		1769		381		2522		155		1173		258		1830	

As in the overall sample, more respondents reported serious health problems on the 5L than on the 3L, and the difference was particularly noticeable in the older age groups. For example, in the oldest age group, only 3.1 % of respondents reported being in very poor health (level 3) in the usual activities dimension on the 3L, compared to 12.4 % reporting level 4 or 5 problems on the 5L. A similar pattern was seen on most of the other dimensions. The differences in the proportions of respondents reporting very poor health between the two versions were statistically significant in all age groups for the mobility and usual activity dimensions (*P* < 0.05). For self-care, the differences between the two versions were statistically significant in all age groups (*P* < 0.05) except the youngest (*P* = 0.06). For the pain/discomfort dimension, the differences between the two versions were statistically significant in all age groups (*P* < 0.05) except the 55–64 years age group (*P* = 0.17). There were no statistically significant differences between the two versions in any age group for the anxiety/depression dimension.

Differences by sex and level of education were also in the expected direction (Table 5), with males and those in the higher educational category reporting better health than females and those without a degree. However, there was very little difference between the two versions of EQ-5D in terms of ability to discriminate between groups based on these two variables.

**Table 5:** Number (percentages) of respondents at level 1 in the EQ-5D-5L profile and EQ-5D-3L profile, by education and sex

EQ-5D dimensions	5L version			3L version			5L version			3L version		
Education and sex	Degree	No degree	*P* value	Degree	No degree	*P* value	Male	Female	*P* value	Male	Female	*P* value
Mobility	181 (85.8 %)	556 (70.8 %)	0.00	1728 (92.1 %)	4288 (79.3 %)	0.00	306 (75.6 %)	431 (72.9 %)	0.35	2697 (83.3 %)	3324 (81.9 %)	0.11
Self-care	203 (96.2 %)	701 (89.3 %)	0.00	1831 (97.6 %)	5058 (93.5 %)	0.00	369 (91.1 %)	535 (90.5 %)	0.75	3060 (94.6 %)	3834 (94.5 %)	0.88
Usual Activities	180 (85.3 %)	580 (73.9 %)	0.00	1726 (92.0 %)	4399 (81.3 %)	0.00	310 (76.5 %)	450 (76.1 %)	0.88	2778 (85.9 %)	3352 (82.6 %)	0.00
Pain/Discomfort	145 (68.7 %)	437 (55.7 %)	0.00	1451 (77.3 %)	3393 (62.7 %)	0.00	253 (62.5 %)	329 (55.7 %)	0.03	2246 (69.4 %)	2602 (64.1 %)	0.00
Anxiety/Depression	167 (79.2 %)	590 (75.2 %)	0.23	1591 (84.8 %)	4236 (78.3 %)	0.00	320 (79.0 %)	437 (73.9 %)	0.07	2701 (83.5 %)	3130 (77.1 %)	0.00
Sub-total	211 (21.2 %)	785 (78.8 %)		1877 (25.7 %)	5417 (74.3 %)		405 (40.7 %)	591 (59.3 %)		3236 (44.4 %)	4058 (55.6 %)	

**Fig. 1:**
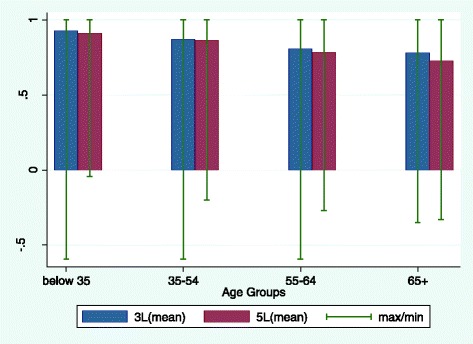
EQ-5D Index scores by age group for the two versions of EQ-5D

**Fig. 2:**
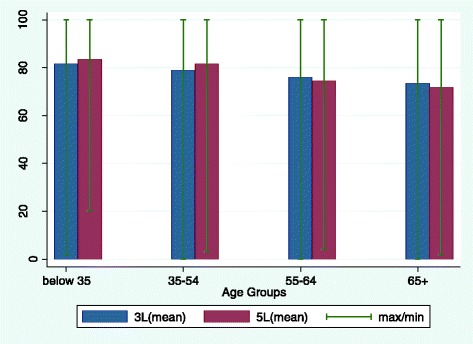
EQ-VAS scores by age group for the two versions of EQ-5D

The distribution of EQ-5D Index and VAS scores are shown by age group in Figs. 1 and 2, respectively. Figure 1 shows that EQ-5D Index scores decreased with age on both versions of the instrument. The decreases in score between each consecutive age group were statistically significant in all cases for both versions of the EQ-5D (*P* < 0.05). EQ-5D-3L Index scores were higher overall than those for the 5L version for all four age groups, though the difference was only statistically significant in the oldest age group (*t* = 2.95 with *P* < 0.05). The difference in mean Index score between the youngest and oldest age groups was also slightly greater using the 5L (0.16 for the 3L vs 0.18 for the 5L). The EQ-VAS showed a similar trend (Fig. 2), with EQ-VAS scores decreasing by age. The decreases between age groups were statistically significant in all cases on the 3L (*P* < 0.05), but on the 5L the reduction in VAS scores was only statistically significant between the 35–54 and the 55–64 year age groups (*P* < 0.05). However this is likely to be due to sample size as the between group differences were at least as large on the 3L as on the 5L. The difference in VAS scores between the oldest and youngest groups was also larger with the 5L than the 3L, though the difference between the two versions was minimal.

**Fig. 3:**
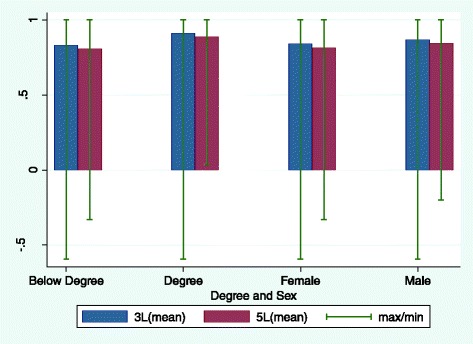
EQ-5D Index scores by education and sex for the two versions of EQ-5D

**Fig. 4:**
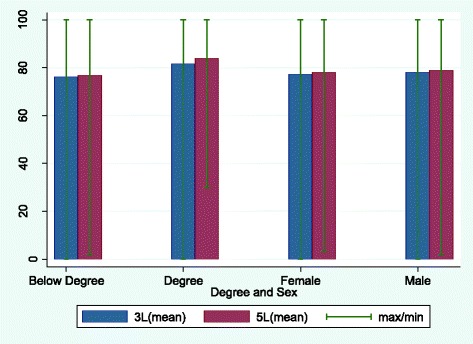
EQ-VAS scores by education and sex for the two versions of EQ-5D

EQ-5D Index and EQ-VAS scores are shown in Figs. 3 and 4 by level of education and sex, respectively. The two scores show a similar pattern to that seen for the EQ dimensions, with degree holders reporting better health than non-degree holders (*P* < 0.05 for EQ-5D-5L Index, EQ-5D-5L VAS, EQ-5D-3L Index and EQ-5D-3L VAS), and male respondents having higher scores (better health) than female respondents (difference only statistically significant for EQ-5D-3L Index, at *P* < 0.05). Again, the EQ-5D Index leads to slightly higher scores with the 3L version, though the difference in scores between categories (degree-no degree, male–female) was very similar on both Indices and VAS.

## Discussion

The aim of this study was to compare the performance of the 3L and 5L versions of EQ-5D in representative samples of the English general public. We found that a) the ceiling effect was considerably reduced using the 5L version of EQ-5D, and that the reduction was particularly noticeable in older age groups; b) the EQ-5D-5L provided a richer description of health status, with just three EQ-5D-3L health states accounting for 75 % of the sample compared to 12 states using the 5L; c) higher proportions of respondents reported serious health problems using the 5L; d) both versions showed poorer health-related quality of life (HRQOL) in older respondents, females, and those in a lower educational category; e) the EQ-5D-5L descriptive system discriminated better across age groups than the 3L, though not by sex or educational level.

The reduction in ceiling effect using the 5L has been found in other studies which compared the 3L and 5L versions [25, 26], but they were performed in patient groups. Only the Craig et al. study (2014) and this study have compared the two versions in general population samples. Our findings suggest that the 5L version provides a fuller and more detailed picture of population health status. Of note was the fact that the 5L showed more respondents suffering severe health problems, presumably because of the greater descriptive richness of the 5L. In the 3L, respondents must choose between ‘some’ problems and ‘unable to whereas in the 5L they can choose between ‘moderate’, ‘severe’ or ‘unable to’.

Comparing our findings to Craig et al. (2014), both studies found that the percentage of respondents reporting levels 4 and 5 in the 5L version of EQ-5D was higher than the percentage reporting level 3 in the 3L version. Based on these results, Craig et al. (2014) suggested that the 5L led to a greater frequency of health problems being reported (because of the lower ceiling effect), but that those health problems tended to be less severe, as the ‘unable to’ or ‘extreme’ category was used less frequently on the 5L, at least in the usual activities, pain/discomfort and anxiety/depression dimensions. Our findings were similar in this regard, but we would argue that, instead of showing fewer respondents with severe health problems, as suggested by Craig et al., the 5L actually reflects a greater percentage of respondents with severe health problems as we consider respondents checking either the ‘severe’ or ‘unable to/extreme’ options on the 5L to fall into that category. Van Hout et al. (2012) provides a cross tabulation for EQ-5D-3L and EQ-5D-5L responses by dimension. Their results suggest that in pain/discomfort dimension and anxiety/depression dimension majority of respondents who reported level 3 (extreme problems) in the EQ-5D-3L reported level 4 (severe problems) in the EQ-5D-5L. The same pattern was observed in Craig et al. (2014). On the other hand, Craig et al. (2014) found a lower percentage of respondents with self-reported full health ‘11111’ on the 3L (44 %) and 5L (35 %) than we did (56.2 % for the 3L and 47.6 % for the 3L). This is somewhat surprising given that the Craig et al. sample was somewhat younger and had a higher proportion of males, but it may be due to some extent to the different methods for data collection, i.e. the use of online data collection in Craig et al. (2014) compared to face-to-face interviews in both of the surveys used in the present analysis.

As expected, both versions of EQ-5D discriminated satisfactorily between groups defined according to their socio-demographic characteristics. Previous research has shown that older age groups, females, and those in lower educational categories report poorer HRQOL [27–29] and the current results confirm those findings. However, we were also interested in whether the two versions of EQ-5D were equally able to discriminate between groups according to their socio-demographic characteristics. In this case, we found that there were notable differences between the 3L and 5L when comparing between different age groups, with, for example, a difference of 35.6 % points between the oldest and the youngest age groups in the proportion of respondents reporting no problems with usual activities using the 5L compared to a difference of 20.6 % points using the 3L. A similar pattern, with the 5L suggesting a broader gap in health status between the youngest and oldest age groups than the 3L, was seen across most of the other dimensions, though to a lesser extent on anxiety/depression and not at all on pain/discomfort. There were no appreciable differences between the two versions in terms of their ability to discriminate between groups based on sex or educational level.

The differences between the 3L and 5L in outcomes on the descriptive system across age groups largely disappeared when applying the EQ-5D Index. The fact that we used the Van Hout et al. crosswalk value set to calculate Index values for the 5L may have contributed to this difference in performance between the 5L descriptive system and the Index, as Index values for the 5L are restricted to the range of values in the 3L value set [24]. We also found that 5L Index values were slightly lower than 3L Index values in all age groups, which is likely due to the fact that more respondents report health problems using the 5L.

Results on the EQ-VAS were also very similar between the two versions, which is to be expected given that only relatively minor modifications were made to the version in the 5L. The instructions were modified to make them easier to follow and 5 point numbering was used in the new version compared to 10 point numbering in the 3L. One notable feature of the VAS is digit preference, whereby responses cluster around tens and to a lesser extent fives [30]. This feature is observed on EQ-VAS in both the 5L and 3L versions of the instrument. The two most frequent self-reported EQ-VAS scores in this study were 90 and 80, respectively, in both versions of EQ-5D. The overall distribution of EQ-VAS scores was similar between EQ-5D-3L and EQ-5D-5L: 8 out of the 10 most frequently observed self-reported EQ-VAS scores in the 3L version are reported in the 5L version as well. Additional details of the prevalence of self-reported EQ-VAS scores on the EQ-5D-3L and EQ-5D-5L can be obtained upon request from the authors.

### Limitations

One limitation of the current study is the difference in the size and characteristics of the samples used for the 5L and 3L data. The smaller sample size for the 5L data would have led to less statistical power for some of the analyses performed, for example when comparing rates of problem reporting in the different dimensions or when comparing Index and VAS scores across different socio-demographic categories. Likewise, the differences in socio-demographic and health characteristics may have contributed to some of the differences between EQ versions reported here. For example, the slightly higher proportion of females and those in the lower educational category in the 5L sample may have led to more reporting of problems in that group. As the differences between the samples were small (differences between the two samples for all categories are under 5 %) and we considered that they were unlikely to affect results to a great extent, we did not adjust for them in the statistical analysis. A more complex approach that adjusted the samples to be nationally representative would likely also introduce a greater level of uncertainty around any point estimates.

Second, as noted in the Methods section, there is a mismatch between the value sets we used (i.e. EQ-5D-3L value set for UK and the crosswalk value set for UK) and the profile data analysed. Both the 3L and 5L value sets were developed based on valuation studies in the UK, whilst the profile data was from population surveys in England in both cases. However, value sets for England alone were not available when this study was performed and we considered that the best available option was to use the UK value set. The difference should be borne in mind when interpreting these results. It would also have been preferable to use a 5L-specific value set for this analysis rather than values obtained using the crosswalk approach, however, the 5L value set for England was not available at the time this analysis was performed.

Finally, our categorization of educational level in the present analysis was somewhat crude and a more refined categorization might have provided additional information on the ability of both versions of EQ-5D to discriminate across categories on this variable. However, we felt that the categorization used was sufficient for this initial examination of the discriminatory power of the 3L and 5L versions of EQ-5D.

## Conclusions

This study compared the performance of the 3L version of the EQ-5D to the newer, expanded five level version, in measuring the HRQOL of the general population in England. Overall, the 5L provided a richer description of health status in the population, and improved the instrument’s measurement properties, by reducing the ceiling effect and improving discriminatory power, at least by age group. It is likely to be the more useful of the two versions for inclusion in health population surveys.

## Appendix 1

**EQ-5D-5L descriptive system**

**EQ-5D-3L descriptive system**

## Appendix 2

**The EQ-VAS in EQ-5D-5L** [31]

**The EQ-VAS in EQ-5D-3L** [32]

## Abbreviations

VAS: Visual Analogue Scale
HSE: Health Survey for England
PAF: Postcode Address File
PROMs: Patients Reported Outcome Measures
HRQOL: Health-related quality of life
